# Case of Tumor in the Anterior Wall of the Uterus, and Extensive Disease of the Right Ovary

**Published:** 1853-01

**Authors:** S. G. Bailey

**Affiliations:** Buffalo, N. Y.


### ART V. — *Case of Tumor in the anterior wall of the Uterus, and extensive disease of the right Ovary.* By S. G. Bailey, M. D., Buffalo, N. Y.

The following case may be interesting, in connection with others, of diseases of these organs; though, alone, it might not possess any particular interest. The subject of it was ill for the last ten years of her life. Her history during that period is given as correctly as it could be gathered by careful inquiry—since January 8, 1852, during my attendance upon the patient,—up to that time when she first came under my observation. It was impossible to make a fully satisfactory diagnosis, which I believe is often true in these cases. The early life, and early married life, of the patient was healthy. She was a farmer's daughter in Canada — and says she has often spent whole days in the hay-field at hard work, as was the custom there in her young days.

Her parents were healthy. Does not know that her mother ever suffered in the least from uterine diseases or derangements. Patient was married at the age of seventeen years. Has had three children, the youngest now twelve years old, the eldest seventeen. After the second child, thinks she had an abortion, and was not well for near two years, when her health improved and she became pregnant with the third and last child. Does not recollect anything unusual in relation to her health during her last pregnancy. Enjoyed good health after the birth of her child for about two years, when she commenced to have the floodings that continued during the remainder of her life. From these floodings which occurred every month, and in increasing severity, she became so prostrated in about two years, that alarming faintings began to be experienced at these periods. The flow usually continued four or six days, by which time she would be compelled to keep her bed, which, aided probably by the exhaustion, would check the discharge, and another week in bed would suffice for her to regain sufficient strength to begin to get about again. A considerable strength would be regained between the periods, though subject to a constant slight discharge, a little colored, during the whole time. Never experienced much pain. Appetite always good; bowels regular. Has been troubled with a dry cough most of the time for the last five years, but it is exclusively sympathetic, and much increased by irritating the os uteri.

I first saw patient January 8, 1852. Within the last few months previous to that time, had been getting much worse. The discharge constant, profuse, and offensive, and much increased at the beginning of her monthly periods, but one day now of the flooding will completely prostrate her, and the discharge abates.

Has brown hair, blue eyes, blanched lips, and pearly skin. Is much emaciated. Thoracic organs are all good, organically and functionally. Examination by speculum presents the anterior lip of the os uteri much thickened and enlarged; the orifice and passage ulcerated as far in as could be examined. The ulceration I cauterized with Nit. Argt. There is now a dark-colored and offensive discharge, which the speculum shows to come from, or through the uterus. Griffith's myrrh mixture and quinia were prescribed, for the system generally, and an injection of solution of Nit. Argt., locally. This treatment was continued for a month, occasionally omitting the quinia, in which time both strength and health had improved much. The flowing at the next period was less and more natural, and the discharge during the interval less and more healthy. She continued to improve till April 1st, though medicine was not constantly used, when, feeling so much better the medicine was omitted entirely, and I did not hear from her again until about the middle of May. The medicine was stopped contrary to my advice. May 1st, patient moved her residence, and thinks she over-worked. From this time she did not feel so well, and about the middle of the month the menses appeared, and the flow was again profuse. All the worst symptoms that she had ever had before, now appeared again, and her prostration was extreme. Large accumulations of coagula were discharged and the uterine organs were, from external appearances, increased in size with some tenderness manifest on pressure over them. About the 20th of May when the urgent symptoms had subsided a little, I discovered on more particular examination externally, a hard, round, movable tumor, which seemed to occupy exactly the position of the uterus. It was about the size and feel of the uterus, when contracted down, directly after delivery at childbirth. Was some tender upon pressure, but not remarkably so. In whatever position the patient placed herself the tumor would float upon the surface, or present itself at the most elevated point of the more yielding parts of the lower portion of the abdomen. Examination per vagina, afforded no assistance in the diagnosis, of the seat or character of this tumor.

It was discovered by the patient herself several days before I first noticed it, but it was, when first felt by patient, the full size that it ever attained. It appeared to her to have grown in a night. She did not seem to think it of consequence enough to call my attention to it, so accustomed had she become to afflictions, and changes from better to worse, and worse to better during the last ten years of her life. Of the diseased condition of the ovary we had no other indication than the discharge. There had been no hectic fever, or chills, or any other constitutional manifestations of so extensive a diseased condition as was revealed by post mortem examination. The local symptoms were fully sufficient to indicate extensive disease somewhere. The discharge per vagina had again become very offensive and irritating, of a dark color and thin in consistency. The treatment was resumed, as employed before, but with little satisfaction. In addition, some discutients were used over the tumor externally, though entirely ineffectual for good. About June 20th I asked counsel, and Dr. White saw the patient with me. We did not locate the tumor definitely in our diagnosis. In order to determine whether the tumor was within the cavity of the uterus or not, the os uteri was largely dilated by sponge tents. We found only some small warty excrescences, three or four attached near the fundus, and not larger than a large, long bean. They could be easily felt and distinguished by the exploration of the cavity of the uterus by the finger. These could not be the tumor felt externally, neither was it possible to tell now whether this tumor was made, or formed in the substance of the walls of the uterus, or one of the ovaries. Full examination, and exploration of the cavity of the uterus by the uterine sound, enabled us to determine that the tumor was not within its cavity.

From this examination, too, we were quite sure that it must be where the post mortem disclosed it, but that was not *clearly* evident.

Tonics, alteratives, and discutients, were from this time onward faithfully employed, which seemed the only means admissible for application in the case.

Anæsthetics and anodynes were attempted for the relief of pain, which at times of late had become very severe, but could not be tolerated.

She steadily declined, and died August 18, 1852.

Post mortem thirty-six hours after *death*. On opening the abdomen we found the anterior wall of the uterus entirely occupied by, and transformed into a hard, round, cartilaginous tumor, about 3½ or 4 inches in diameter; the right ovary enlarged to about the same dimensions and excavated; so that its walls were quite thin, by an ulcerative action, presenting a ragged, rough, and black surface, internally, and secreting a thin, black matter.

The anterior lip of the os uteri was a little enlarged, though not so much so as upon my first examination in January, on first seeing the patient. The warty excressences were found in the cavity of the uterus, as felt after the dilation of the os by the sponge tent. They were probably stimulated by the irritating discharge from the ovary, pathologically, possessing no further interest.

Several interesting questions arise in the consideration of this case:

*First.* The duration of the diseased action in the ovary; its origin and cause.

*Second.* Had this tumor grown entirely during the time from the last of February till the middle of May, or had it been a long time during the eight years forming, and growing slowly, and then thus suddenly developing itself in the space of three months? Nothing of the kind could be discovered upon my first seeing the patient.

*Third.* What influence had the tumor and the diseased ovary upon each other? The history of the case seems to indicate that the ulceration in the ovary must have been in existence from the first, producing those hæmorrhages from its surface indicated by the discharge of coagula at every menstrual period.

*Fourth.* Would it be possible for pregnancy to take place after the development of the disease in the ovary?

This question is worthy of consideration from the fact that the last pregnancy occurred after a supposed abortion, which, under the circumstances, might not have been such, the symptoms of which being similar to those often experienced afterward when no such condition could reasonably be supposed to exist. The opposite ovary, too, was perfectly healthy.

The diseased ovary, if distended, by filling its cavity with fluid, would be about the size of the hard tumor in the wall of the uterus, from 3½ to 4 inches in diameter.

